# Mouse IgG2a Antibodies Specific for the Commensal *Streptococcus mitis* Show Stronger Cross-Reactivity with *Streptococcus pneumoniae* than IgG1 Antibodies

**DOI:** 10.1155/2019/7906724

**Published:** 2019-09-10

**Authors:** Sudhanshu Shekhar, Rabia Khan, Ata Ul Razzaq Khan, Fernanda Cristina Petersen

**Affiliations:** Institute of Oral Biology, Faculty of Dentistry, University of Oslo, Oslo, Norway

## Abstract

Here we show that mouse IgG2a and IgG1 antibodies specific for the commensal *Streptococcus mitis* cross-react with pathogen *Streptococcus pneumoniae* serotypes 2 and 4, although the cross-reactivity conferred by IgG2a is stronger than that by IgG1 antibodies. These findings may be important for understanding the *S. mitis*-induced IgG isotype responses and have consequences for the development of an effective pneumococcal vaccine.

## 1. Introduction

*Streptococcus pneumoniae*, a gram-positive bacterium that resides in the human upper respiratory tract, causes several diseases worldwide, such as pneumonia, sepsis, meningitis, and otitis media [1]. Due to differences in polysaccharide capsules, *S. pneumoniae* has been classified into more than 90 serotypes, which reflect different antigenicity and geographical distribution [2]. Current pneumococcal vaccines are effective against only those *S. pneumoniae* serotypes that are included in vaccines [2]. This underscores the exploration of alternative prophylactic strategies that provide protection against all serotypes. In recent years, *Streptococcus mitis*, a commensal that is abundantly present in the oral cavity and shares phylogenetic, antigenic, and ecological characteristics with *S. pneumoniae*, has appeared as an interesting vaccine candidate that holds the potential to confer optimal immunity to pneumococcal infections [3, 4]. Although recent studies have indicated a crucial role for IgG antibodies in defense against pneumococcal infections, it is unclear as to how *S. mitis* stimulates the host's immune system to generate humoral responses [5]. Emerging evidence reveals that rabbits immunized with *S. mitis* generate IgG antibodies that are reactive with both *S. mitis* and *S. pneumoniae* [3]. In line with this, we have recently shown that mouse IgG antibodies specific for *S. mitis* exhibited cross-reactivity toward *S. pneumoniae* strains D39 (serotype 2) and TIGR4 (serotype 4) [4]. Murine IgG antibodies are heterogeneous consisting of four subclasses (IgG1, IgG2a, IgG2b, and IgG3). Out of which, IgG2a and IgG1 antibodies play an important role in pathogen defense by opsonization/complement fixation and immune effector functions, respectively [6, 7]. Furthermore, nasopharyngeal colonization of mice with *S. pneumoniae* triggers a significant rise in the levels of antigen-specific IgG2a and IgG1 antibodies [8]. It however remains unknown whether *S. mitis*-specific IgG antibody responses that are cross-reactive to *S. pneumoniae* are biased to an IgG subclass, such as IgG2a and IgG1. Therefore, the goal of this study was to explore whether (1) *S. mitis* induces the production of antigen-specific IgG1 and IgG2a antibodies and (2) these antibodies cross-react with *S. pneumoniae* serotypes. To accomplish this, we intranasally immunized mice with *S. mitis* and examined the IgG reactivity to *S. mitis* and *S. pneumoniae* by Western blotting and ELISA. Our findings provide new knowledge that *S. mitis* elicits the production of IgG isotypes that show cross-reactivity with *S. pneumoniae*. This may be important for understanding the commensal bacteria-host interplay and designing commensal-based vaccines against pneumococcal infections.

## 2. Material and Methods

### 2.1. Bacterial Culture and Lysis

The bacterial strains used in this study included *S. mitis* CCUG31611 (type strain, equivalent to NCTC12261), *S. pneumoniae* D39 (serotype 2), and *S. pneumoniae* TIGR4 (serotype 4). All strains were cultured, harvested, and stored as described previously [3]. To lyse the bacterial cells, a Precellys lysing kit and a homogenizer (Precellys 24, Bertin Instruments) were used as per the manufacturer's instructions. After homogenization, the bacterial cell lysate was centrifuged at 1000g for 5 min at 4°C and the supernatant was collected and stored at −80°C for further use. The total protein in the cell lysate was quantified using the BCA Protein Assay Kit (Thermo Fischer Scientific).

### 2.2. Mouse Immunization

CD1 mice used in this study were females of 6-8 weeks age. These mice were specific pathogen free (SPF) and housed in a minimal disease unit at the animal facility at Oslo University Hospital, Rikshospitalet, Oslo, Norway. All animal experiments were approved by the Norwegian Food Safety Authority, Oslo, Norway (project license numbers FOTS 8481 and 10515), and performed in accordance with the guidelines of the Norwegian Animal Welfare Act (10 June 2009, no. 97), the Norwegian Regulation on Animal Experimentation (REG 2015-06-18-761), and the European Directive 2010/63/EU on the Protection of Animals Used for Scientific Purposes. The mice were allowed 1-week acclimatization before experiments were started. The mice were intranasally immunized with 5 × 10^7^ colony-forming units (CFU) of *S. mitis* in 20 *μ*l of PBS or 20 *μ*l of PBS (sham) for each mouse at days 0, 14, and 21. The mice were euthanized at day 7 after the last immunization to collect the blood via intracardiac injection. The freshly isolated blood was kept at 4°C for 1 hour and then centrifuged at 1000g for 5 minutes. The supernatant sera were collected and preserved at -80°C in a refrigerator for further analysis.

### 2.3. Western Blotting

As described previously [4], SDS-PAGE-separated proteins were transferred from gradient gels (4–20%) to nitrocellulose membranes using a constant voltage of 100 V for 2 h using the Bio-Rad Midi PROTEAN Western blotting module according to the manufacturer's instructions (Bio-Rad, CA, USA). The membranes were blocked with blocking solution (Tris-buffered saline (TBS) (pH 7.4) containing 0.1% Tween 20 (Sigma-Aldrich) and 5% BSA) for 1 h at room temperature, washed three times with TBS-Tween, and incubated overnight with antisera at 4°C. The antisera were diluted to 1 : 1000. Following incubation, the membranes were washed and finally incubated with AP-conjugated anti-mouse IgG2a or IgG1 secondary antibodies and later developed with the substrate 5-bromo-4-chloro-3-indolyl phosphate (BCIP)/nitro blue tetrazolium (NBT) (Sigma-Aldrich).

### 2.4. Whole-Cell ELISA

To determine antibody levels in mouse serum samples, a whole-cell ELISA was used as described previously [3]. In brief, each well of a 96-well plate (Maxisorb, Nunc, Thermo Scientific) was coated overnight with 100 *μ*l of bacterial suspension (OD600 0.5), which was washed and then fixed with 10% formalin. The plate was washed and blocked with a blocking buffer (PBS+0.05% Tween+1% BSA) and incubated for 1 h at 37°C. Antisera were diluted (1 : 100) and added to wells in duplicate and incubated for 2 h at room temperature before the addition of the anti-mouse IgG2a/HRP or anti-IgG1/HRP secondary antibody (1 : 10,000) followed by incubation for 2 h at room temperature. The plates were washed, and 100 *μ*l of TMB substrate (Thermo Fisher Scientific, Rockford, IL, USA) was added to each well. The plate absorbance was measured by reading the plates at 450 nm using a Multi-Mode reader (BioTek™ Cytation™ 3; Thermo Fisher Scientific).

### 2.5. Statistics

Unpaired Student's *t*-test was used to compare two groups of mice (GraphPad Prism Software, version 8, GraphPad, San Diego, CA, USA). A *p* value less than 0.05 was considered significant.

## 3. Results

**Figure 1:**
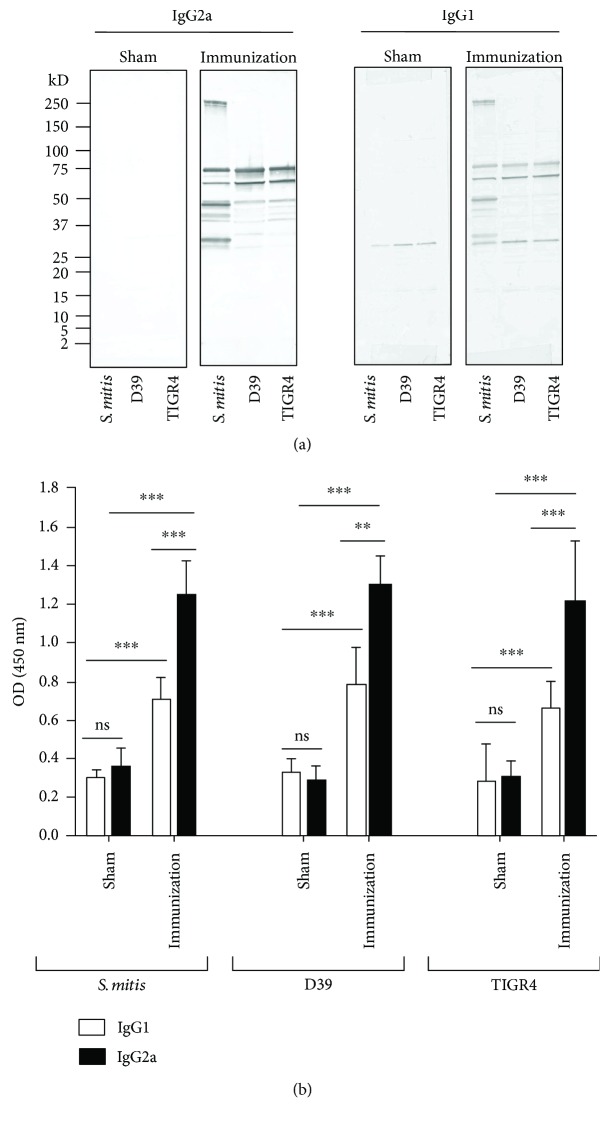
IgG2a and IgG1 antibodies from the *S. mitis*-immunized mice cross-react with *S. pneumoniae.* CD1 mice were intranasally immunized with live *S. mitis*, and sera were collected to analyze antibody responses to *S. pneumoniae* strains D39 (serotype 2) and TIGR4 (serotype 4). (a) Cross-reactivity of *S. mitis*-specific IgG isotypes with *S. pneumoniae* serotypes by Western blotting. Each lane was loaded with 50 *μ*g of protein from the indicated bacterial species. The sera were diluted to 1 : 1000. (b) Levels of antibodies reactive to *S. mitis* and *S. pneumoniae* serotypes by the whole-cell ELISA. The sera were diluted to 1 : 100. Data are shown as mean ± SD and pooled from two independent experiments with 4 mice in each group. Unpaired Student's *t*-test was used to compare the levels of antibodies between sham-treated and immunized mice. ^∗∗^*p* < 0.01; ^∗∗∗^*p* < 0.001. ns: not significant; D39: *S. pneumoniae* D39; TIGR4: *S. pneumoniae* TIGR4.

Our Western blotting showed that serum IgG2a and IgG1 antibodies from the *S. mitis*-immunized mice cross-reacted with multiple proteins of *S. pneumoniae* D39 and TIGR4. This was reflected by several visible bands that were between 25 and 75 kD (Figure 1(a)). Two prominent bands were present at around 250 and 25 kD in the membrane lane loaded with *S. mitis*, but not *S. pneumoniae* serotypes (Figure 1(a)). Compared to IgG2a, IgG1 antibodies from the immunized mice showed weaker reactivity toward *S. mitis* and *S. pneumoniae* (Figure 1(a)). However, IgG2a and IgG1 antibodies from the mice inoculated with PBS (control mice) gave rise to few faint bands (Figure 1(a)). Of note, our data also reflected similar findings in the nasal wash and bronchoalveolar lavage of immunized mice (data not shown). We further measured the levels of serum IgG2a and IgG1 antibodies reactive to *S. pneumoniae* using the whole-cell ELISA. The *S. mitis*-immunized mice displayed significantly higher levels of IgG2 antibodies reactive to *S. mitis* and *S. pneumoniae* D39 and TIGR4 than IgG1 antibodies (Figure 1(b)). Taken together, these findings reveal that following mucosal immunization with *S. mitis*, mice generate enhanced IgG2a and IgG1 antibody responses that cross-react with *S. pneumoniae* serotypes, although the IgG2a responses are stronger than IgG1 responses.

## 4. Discussion

In this study, we have shown that serum IgG2a and IgG1 antibody isotypes induced in response to immunization of mice with *S. mitis* cross-react with *S. pneumoniae* serotypes and that the cross-reactivity induced by IgG2a is stronger than that by IgG1 antibodies. The mouse IgG2a antibody is considered a functional equivalent of human IgG1 antibodies, which constitute the most abundant among all human serum IgG isotypes [9, 10]. Immunization of human infants with pneumococcal conjugate vaccines has been reported to result in rises in IgG1 antibodies and opsonophagocytosis of antibodies to *S. pneumoniae* capsular polysaccharides [11]. In line with these findings, our current study has shown a significant increase in *S. mitis*-specific IgG2a antibodies reactive to *S. pneumoniae* serotypes 2 and 4, which hints at the implication of this antibody isotype in *S. mitis*-mediated protective immunity to *S. pneumoniae*. On the other hand, mouse IgG1 antibodies, which represent the equivalent of human IgG4 antibodies that play a role in defense against *S. pneumoniae*, have been shown to increase in response to nasopharyngeal colonization by *S. pneumoniae* [8, 12]. This is in accordance with our results that the *S. mitis* immunization gives rise to enhanced IgG1 responses reactive to *S. pneumoniae*. However, the cross-reactivity induced by IgG1 antibodies was weaker than that by IgG2a antibodies. Overall, these data add a value to our previous study [4] by providing information on the differential capabilities of *S. mitis*-specific IgG1 and IgG2a antibody responses to *S. pneumoniae*. Therefore, *S. mitis*-based vaccination protocols should also consider the isotypic distribution of antibodies.

## 5. Conclusion

Our findings shed light on the relative capacity of the IgG2a and IgG1 antibodies, which are specific for *S. mitis*, to induce cross-reactivity to pneumococcal serotypes 2 and 4. This may be important for understanding the *S. mitis*-induced IgG isotype responses that cross-react with *S. pneumoniae* and have consequences for effective pneumococcal vaccine development. Future studies are required to explore which IgG isotype plays a dominant role in conferring protection against *S. pneumoniae*.

## Data Availability

The Western blotting and ELISA data used to support the findings of this study are included within the article.
